# Chronic Hand Swelling and Dactylitis in Leprosy: A Case Report and Review of the Literature

**DOI:** 10.7759/cureus.13451

**Published:** 2021-02-20

**Authors:** Sonia Gupta, Changzhao Li, Vinay Kumar Thallapally, Poonam Sharma, Joseph Nahas

**Affiliations:** 1 Internal Medicine, Creighton University, Omaha, USA; 2 Pathology, Creighton University, Omaha, USA; 3 Rheumatology, Creighton University, Omaha, USA

**Keywords:** leprosy, erythema nodosum leprosum, rheumatism, autoantibodies

## Abstract

Leprosy is an infectious disease that is associated with various types of presentations. Diagnosis of the disease can be tricky in cases of atypical presentations. We report a unique case of leprosy characterized by chronic hand swelling, dactylitis, and seropositive laboratory markers, which was diagnosed in a rheumatology clinic.

## Introduction

Hansen’s disease (leprosy) is an infectious disease that affects the skin, peripheral nerves, upper respiratory tract, and eyes. Musculoskeletal involvement is common and the prevalence of arthritis in leprosy ranges from 1-78% [1]. Hence, it is important for rheumatologists to be familiar with the various aspects of this condition. Leprosy can have autoantibodies, and the topic of the prevalence of autoantibodies in patients with leprosy is a controversial one. It is usually diagnosed clinically. Diagnosis can be challenging in cases with non-specific symptoms, especially rheumatic symptoms, which are often referred to a rheumatology clinic. Through this case report, we attempt to highlight and analyze leprosy with rheumatologic symptoms as the initial presentation.

## Case presentation

**Figure 1:**
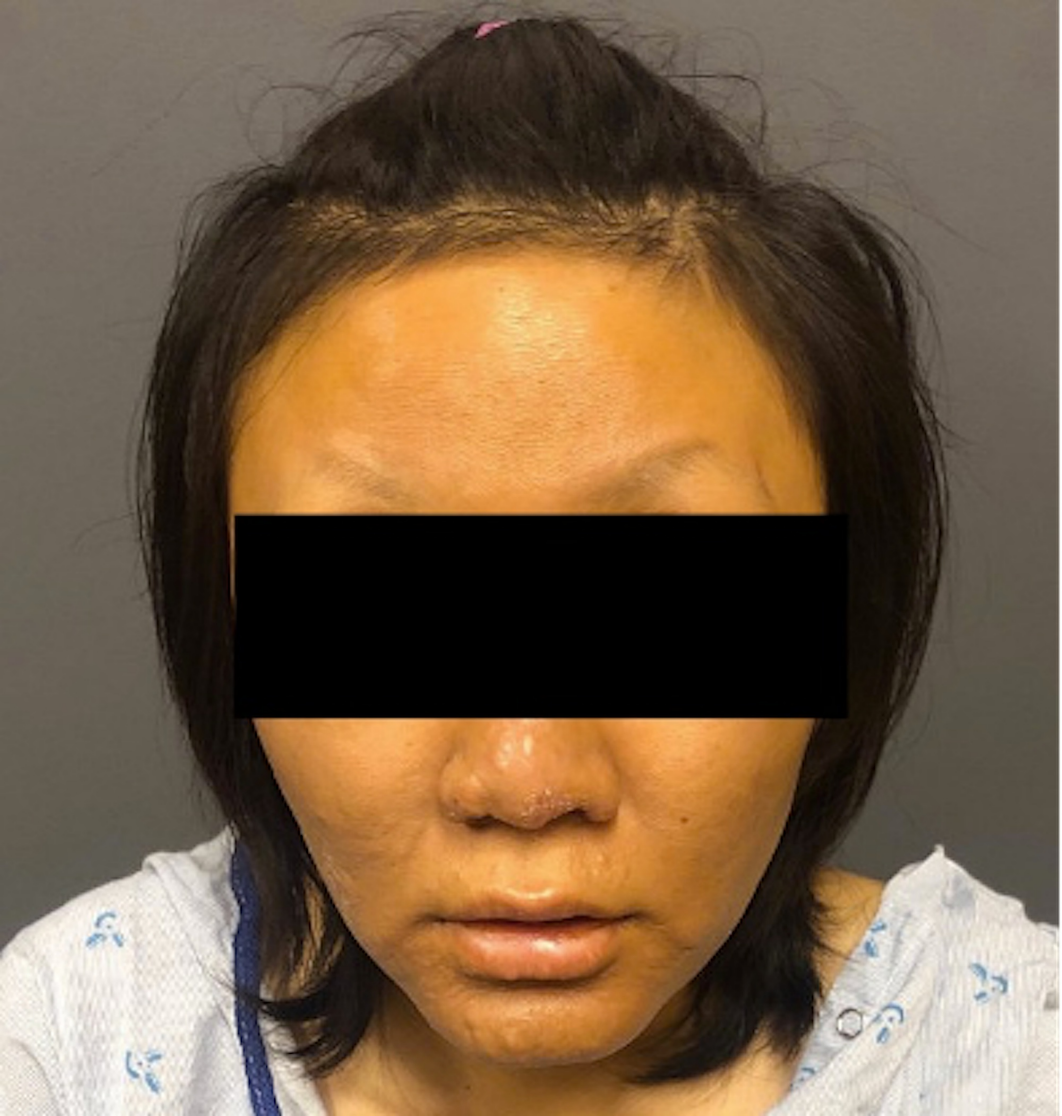
Picture showing facial swelling and loss of lateral eyebrows bilaterally

**Figure 2:**
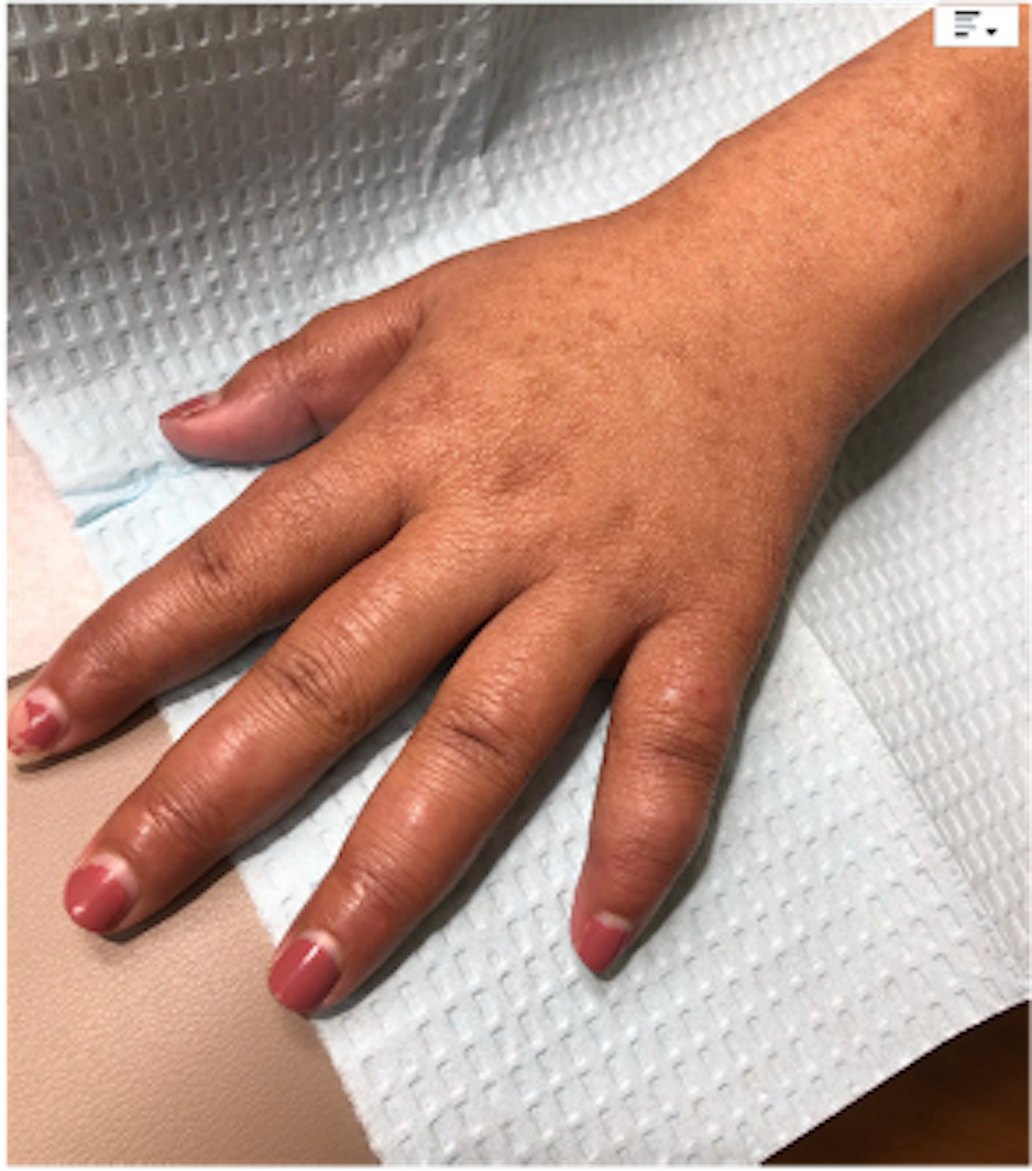
Swelling of joints of the left hand

A 24-year-old female, an immigrant, presented with swelling of the face and bilateral hands of one month's duration. She had initially visited her primary care physician for painful swelling over the small joints of both the hands and feet along with painful rash over the arms and legs of one month's duration. She also had intermittent fever and morning stiffness. Laboratory workups were ordered with autoimmune disorders as the main differential. Blood workup showed normocytic normochromic anemia. Inflammatory markers including erythrocyte sedimentation rate (ESR) and C-reactive protein (CRP) were found to be elevated. Regarding autoantibodies, anti-nuclear antibody (ANA), anti-cyclic citrullinated peptide (CCP), anti-cardiolipin (ACL), and beta 2 glycoprotein were positive, while rheumatoid factor (RF), SS-A/Ro autoantibody, SSB/La autoantibody, anti-Smith (Sm) antibody, anti-double-stranded DNA (anti-dsDNA) antibodies, anti-Jo-1 antibody, and anti-centromere and perinuclear anti-neutrophil cytoplasmic antibodies (p-ANCA) were negative. Complement 3 (C3) and complement (C4) were normal (Table 1). X-ray of the bilateral hands was unremarkable. The patient was started on oral steroids and referred to a rheumatology clinic. On presentation to the rheumatology clinic, her face, hand, and feet were puffy with tenderness over small joints (Figure 1, Figure 2). There was a vague, ill-defined patch over the forehead and tender hyperpigmented nodules on the arm and legs. Peripheral nerves were not enlarged. Muscle strength was normal. The sensation was decreased on the lateral side of the left hand. She also had blunting of fine touch and pain sensation in the distal extremities. Clinical differential diagnosis included various rheumatologic diseases including rheumatoid arthritis. Skin biopsy of the painful nodules from the arm was performed on the same day.

**Table 1:** Laboratory markers including autoantibodies at the initial encounter ESR: erythrocyte sedimentation rate; CRP: C-reactive protein; ANA: antinuclear antibody; anti-CCP: anti-cyclic citrullinated peptide; ACL: anticardiolipin; RF: rheumatoid factor; anti-Sm: anti-Smith; anti-dsDNA: anti-double-stranded DNA; p-ANCA: perinuclear anti-neutrophil cytoplasmic antibodies; C3: complement 3; C4: complement 4

Variables	At initial encounter	Reference range
White blood count	8.2	4-12 k/uL
Hemoglobin	10.1	12-16 g/dL
ESR	114	0-25 mm/hr
C-reactive protein	13.40	<9 mg/L
Anti-CCP	26	0-19 units
RF	<10	<15 IU/ml
ANA	Positive (titer was not done)	
Anti-dsDNA antibody	1	0-4 IU/ml
Anti Jo-1 immunoglobulin G	<0.2	0-9 IU/ml
Anti-Sm antibody	0	0-40 AU/ml
p-ANCA	Negative	
Anti-beta glycoprotein immunoglobin M	112	<=20 U/ml
Anti-beta glycoprotein immunoglobulin G	<10.1	<=20 U/ml
ACL immunoglobulin G	<9	0-14 units
ACL immunoglobulin M	>150	0-12 units
C3	154	90-180 mg/dl
C4	25	15-40 mg/dl
25-hydroxy vitamin D	24	30-80 ng/dl

**Figure 3:**
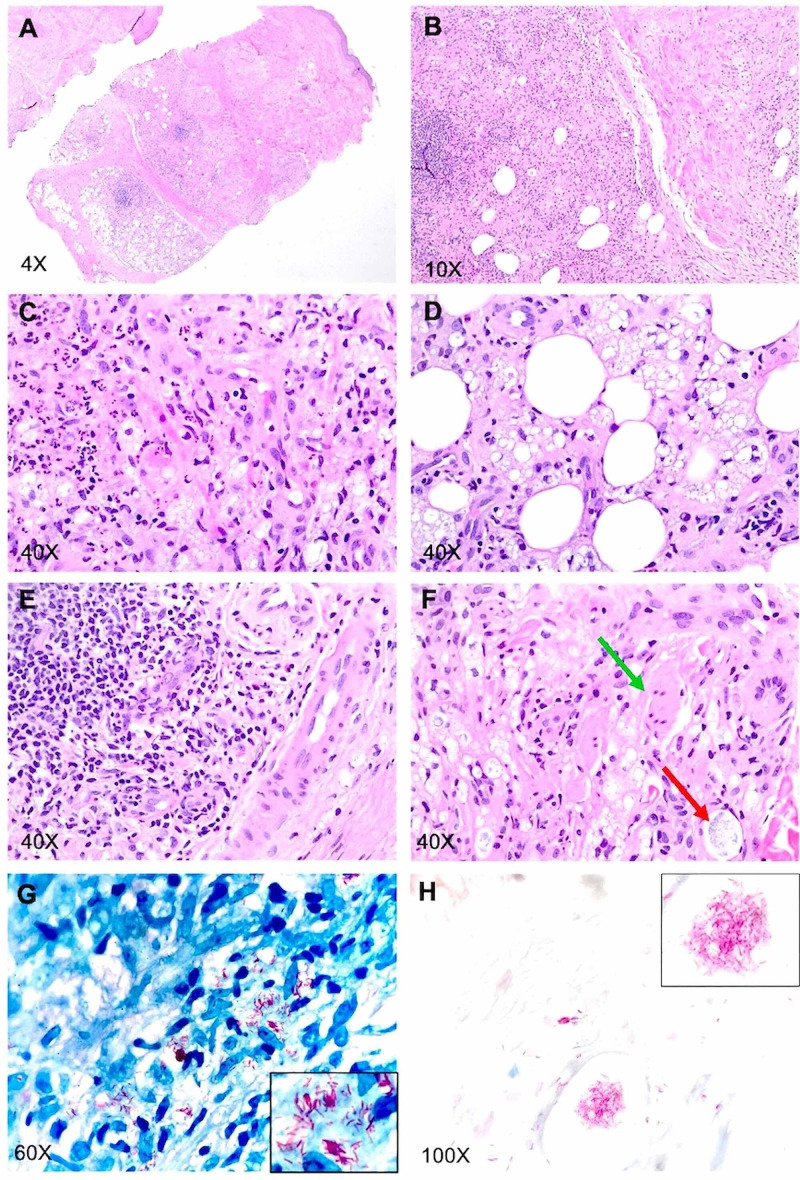
Pictures showing H&E, Fite, and AFB staining of skin biopsy A-F: H&E staining. The red arrow indicates grayish granular material, which was proven to be M. leprae globi following acid-fast stain. The green arrow indicates cutaneous never that is entrapped by foamy macrophages loaded with M. leprae. G: FITE staining. H: AFB staining H&E: hematoxylin and eosin; AFB: acid-fast bacillus

Microscopically, the sections of the skin biopsy from the arm showed skin extending to the subcutaneous tissue. The sections showed acute and chronic inflammatory infiltrates replacing approximately 80-90% of the dermis and extending into the subcutaneous tissue forming lobular panniculitis (Figures 3A, 3B). These infiltrates were composed of aggregates of foamy histiocytes with interspersed lymphocytes, eosinophils, and scattered neutrophils (Figures 3C, 3D, 3E) with surrounding blood vessels, adnexal structures, and cutaneous nerves at all levels of the dermis and subcutaneous tissue (Figures 3D, 3E, 3F). Within the reticular dermis, there were regions where dermal fibrosis intervened (Figures 3A, 3B). However, no vasculitis or fibrin thrombi were observed in the fat lobules and/or in the overlying dermis (Figures 3C, 3E). Histiocytes within the inflammatory infiltrate showed prominent gray-blue granular substance within the cytoplasm (Figures 3D, 3F). Acid-fast Bacilli and Fite stains revealed large numbers of clumped, intact, as well as beaded acid-fast bacilli within vacuolated histiocytes as well as within cutaneous nerves (Figures 3F, 3G, 3H).

**Figure 4:**
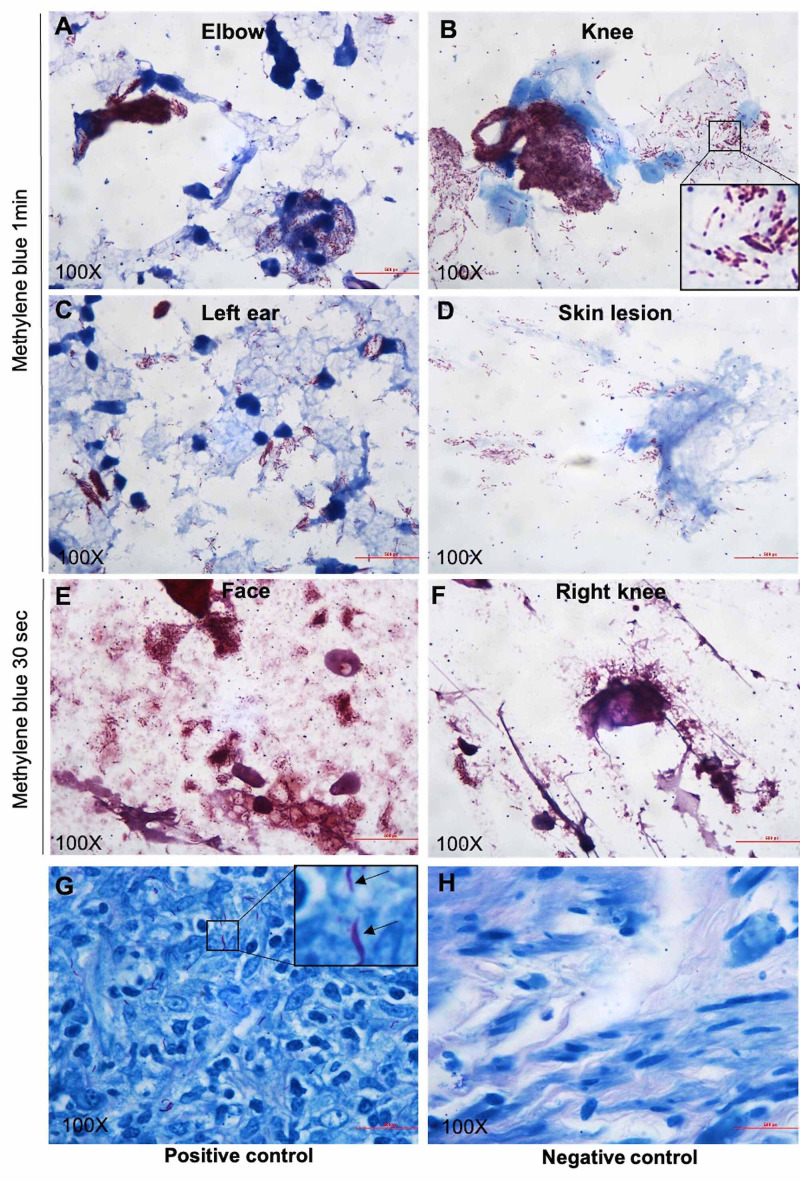
Pictures showing Fite stain of slit skin smear from different locations A-F: slit skin smear. G: positive control. H: negative control

Once the biopsy was back, a slit skin smear was performed in an outpatient setting to rule out leprosy. To assess the bacterial index (BI), air-dried slit skin smears were taken from both ear lobules, knee, forearm, and forehead. The slides were subjected to acid-fast staining with positive and negative controls according to the protocol of “Preparation and Examination of Skin Smears” published on the website of the National Hansen Disease Program (NHDP). To optimize the staining effects, alkaline methylene blue was stained for either one minute (Figures 4A, 4B, 4C, 4D) or 30 seconds (Figures 4E, 4F). The stained smears were examined with a microscope using the oil immersion objective (x100) to determine the total number of bacilli. As shown in Figure 3, a large number of acid-fast bacilli were present in the form of globi (BI: 5+) and were in the smears. Because of the rarity of leprosy in the state of Nebraska, the case was sent to the NHDP for an expert consult and confirmatory molecular test. Polymerase chain reaction (PCR) for Mycobacterium leprae (M. leprae) DNA was positive. The diagnosis of Hansen’s disease [lepromatous (borderline/lepromatous) leprosy, active, consistent with type 2 reaction, erythema nodosum leprosum (ENL)] was finally made by the NHDP. The patient was started on minocycline, moxifloxacin, and rifampin once a month and her swelling has improved after one month of therapy.

## Discussion

The diagnosis of leprosy can be challenging when rheumatological features constitute the initial presentation with minimal skin or nerve involvement. Musculoskeletal and autoantibodies in leprosy can mimic rheumatic diseases. Leprosy may present for the first time with inflammatory arthritis and is mistakenly treated with anti-rheumatic drugs with potentially disastrous consequences [2]. Inflammatory arthritis is commonly seen with lepromatous leprosy with or without lepra reactions [3]. Currently, no definite classification of arthritis exists. However, different forms of articular involvement in leprosy defined in the literature are Charcot joints, septic arthritis, polyarthritis of lepra reaction, and chronic arthritis [1]. The arthritis of lepra reaction is usually acute symmetrical, inflammatory, and polyarthritis affecting small joints of the hands and feet, which presents a picture similar to rheumatoid arthritis. In a cross-sectional study of 24,292 patients with ENL, 24% developed large joint arthritis, and 12% developed joint arthritis. Other extracutaneous manifestations were peripheral edema with 52.4%, dactylitis with 13.7%, and lymphadenitis with 14.7% [4]. The hand swelling in our case was likely due to tenosynovitis caused by M. leprae. The pathogenesis of articular involvement in leprosy is still elusive. Proposed mechanisms include intra-articular immune complex deposition and complement activation that could be involved in ENL-associated arthritis, direct bacilli infiltration of the synovium, or reactive arthritis to mycobacterium antigens [5].

The radiological features can range from normal to subluxation, periostitis, bone resorption, and complete destruction. Clinically painful cutaneous red nodules or plaques can be mistaken as ENL. Systemic symptoms including fever, malaise, and weight-loss as well as synovitis, arthritis, and dactylitis that our patient presented with are common manifestations of ENL [6]. Other rheumatologic manifestations reported include systemic lupus erythematosus (SLE)-like presentation and swollen hand and feet syndrome with remitting seronegative symmetrical synovitis with pitting edema [7], dermatomyositis-like presentation [8], enthesitis, sacroiliitis [1], and isolated tenosynovitis [9].

Leprosy can have autoantibodies and the topic of the prevalence of autoantibodies in patients with leprosy is a controversial one [10]. The frequent autoantibodies in leprosy are RF, ANA, anti-SS-B, antimitochondrial, and antithyroglobulin [11]. A few studies showing the frequency of autoantibodies in leprosy patients are listed in Table 2 [12-17]. The seropositivity can make the diagnosis of leprosy complicated in patients with rheumatology presentation. It is noteworthy that studies have suggested vitamin D deficiency to be associated with increased autoimmune response in leprosy patients [11,13]. The prevalence of antiphospholipid in leprosy is highly variable and is mostly seen in the lepromatous form and as Lucio phenomenon, which clinically presents as painful, stellar lesions with an atrophic scar on the trunk and upper limbs and can mimic vasculitis [17].

ENL is an acute inflammatory reaction to M. leprae antigens, and it commonly occurs after treatment in patients with lepromatous leprosy (LL) or borderline leprosy. Histologically, it is characterized by lobular panniculitis with neutrophilic infiltrates superimposed on chronic multibacillary leprosy [18]. Lobular panniculitis with vasculitis can be seen in rheumatoid arthritis patients and patients with erythema induratum of Bazin (EIB) [18]. However, the neurotropic lymphohistiocytic infiltration with acid-fast bacilli is pathognomonic for Hansen’s disease. In fact, both neurotropic granulomatous and lymph histiocytic infiltrate should point towards Hansen’s disease or at least be considered in the differential diagnosis. Other soft signs of Hansen’s disease include Grenz zone and Virchow cells (foamy macrophages), and the latter was also present in this case [19].

**Table 2:** Table illustrating studies with the percentage of autoantibodies seen in leprosy patients ANA: antinuclear antibody; anti-CCP: anti-cyclic citrullinated peptide; ACL: anticardiolipin; RF: rheumatoid factor; c-ANCA, cytoplasmic anti-neutrophil cytoplasmic antibody; IgM: immunoglobulin M; IgG: immunoglobulin G

Study	Sample size	Anti-CCP	RF	ANA	ACL	c-ANCA	Reference value
Ribiero et al., 2008	185	2.6%	1.6%				12
Guedes-Barbosa et al., 2008	64	16.4%	15%				13
Zavala-Cerna et al., 2012	67	9.3%	41.2%				14
Ribeiro et al., 2012	87			22%			15
Neder et al., 2014	50				IgM; 16%; IgG: 2%		16
Pradhan et al., 2004	75					62.5%	17

In the reported case, some aspects drew our attention. Our patient was a young female with painful swelling of the fingers and positive anti-CCP and ANA that could indicate rheumatoid arthritis as the cause of the illness. Thorough skin and neurological examination, slit skin smear, and biopsy helped in our case diagnosis. Histologically, no vasculitis or fibrin thrombi were observed in the fat lobules and/or in the overlying dermis.

## Conclusions

Our patient initially presented with rheumatologic features and seropositivity, which resulted in her being referred to the rheumatology clinic. Despite the rheumatologic manifestations, neurological findings and slit skin smear turned out to be clues for diagnosis. Several autoantibodies have been mentioned to be positive in such cases in the previously published literature, which was also the case with our patient, and can be misleading. Sometimes, rheumatological manifestation and seropositivity due to leprosy can be easily missed out and overlooked. A thorough clinical evaluation including skin and neurological examination is required to rule out leprosy. We would also like to raise awareness about considering leprosy as a differential diagnosis in arthritis and seropositivity as it will prevent the delay in the diagnosis and potentially devastating consequences with the early administration of immunosuppressants.
